# Dr. Frank Adelbert Jones

**Published:** 1913-04

**Authors:** 


Dr. Frank Adelbert Jones of Rochester, Buffalo 1869, died at his home in Rochester, March 9. He was born in Charlotte, Oct. 23, 1849, his father, Dr. Ambrose Jones, being in practice there. He was the first to die of the members of the Dozen-and-one Club, composed of physicians and their wives and founded 29 years ago. After graduating in medicine he commenced practice in Rochester, but about 1871 moved to Grand Rapids. From 1874 to 1893 he practiced in Charlotte and then moved into the city. He was a Master Mason. He was a member of the A. M. A. and its branches, having been President of the Monroe County Society; he was also a member of the Rochester Academy of Medicine and Pathologic Society and the Central N. Y. Medical Association. He is survived by his widow, formerly Miss Elizabeth R. Wells, to whom he was married in 1869, and by one daughter, Miss Grace L. Jones. Dr. Jones was highly esteemed by his associates and was known as a hard worker, which perhaps accounts for his surprisingly youthful appearance.

